# Ultrasound-guided axial facet joint interventions for chronic spinal pain: A narrative review

**DOI:** 10.1080/24740527.2023.2193617

**Published:** 2023-05-17

**Authors:** Michael J. Wong, Manikandan Rajarathinam

**Affiliations:** Department of Anesthesia & Perioperative Medicine, Schulich School of Medicine and Dentistry, Western University, London, Ontario, Canada

**Keywords:** Ultrasound, intervention, axial, spine, facet joint, chronic pain

## Abstract

**Background:**

Axial facet joint interventions (e.g., medial branch block and radiofrequency ablation, facet joint intra-articular injection) are commonly performed for managing chronic spinal pain. Although traditionally performed with fluoroscopy or computed tomography (CT) guidance, ultrasound-guided techniques have also been developed for these interventions.

**Aims:**

The aim of this study is to present contemporary ultrasound-guided techniques for facet joint interventions and synthesize data addressing their accuracy, safety, and efficacy.

**Methods:**

The PubMed, MEDLINE, CINAHL, Embase, and Cochrane Central Register of Controlled Trials databases were systematically searched for studies of ultrasound-guided facet joint interventions with human subjects from November 1, 1992, to November 1, 2022. Additional sources were drawn from reference lists and citations of relevant studies.

**Results:**

We found 48 studies assessing ultrasound-guided facet joint interventions. Ultrasound guidance for injection of the cervical facet joints and their innervating nerves had favorable accuracy (78%–100%), with lower procedural time compared to fluoroscopy or CT guidance and comparable pain relief. Accuracy with ultrasound-guided lumbar facet joint intra-articular injection (86%–100%) was more reliable than medial branch block (72%–97%); analgesia was comparable to fluoroscopy and CT guidance. In general, these procedures were more challenging for patients with obesity, and deeper structures were more difficult to accurately target (e.g., lower cervical levels, L5 dorsal ramus).

**Conclusions:**

Ultrasound-guided facet joint interventions continue to evolve. Some technically challenging interventions may be impractical for widespread usage or require further technical refinement. The utility of ultrasound guidance with obesity and abnormal anatomy may be reduced.

## RÉSUMÉ

**Contexte:** Les interventions sur l’articulation facettaire axiale (par exemple, le bloc de la branche médiane, l’ablation par radiofréquence et l’infiltration intra-articulaire de l’articulation facettaire sont couramment pratiquées pour traiter la douleur rachidienne chronique. Bien qu’elles soient traditionnellement réalisées sous guidage fluoroscopique ou tomodensitométrique, des techniques guidées par ultrasons ont également été mises au point pour ces interventions.

**Objectifs:** L’objectif de cette étude est de présenter les techniques contemporaines guidées par ultrasons pour les interventions sur les articulations facettaires et de synthétiser les données relatives à ces techniques.

**Méthodes:** Les bases de données MEDLINE, Embase et Cochrane Central Register of Controlled Trials ont fait l’objet d’une recherche systématique d’études portant sur des interventions sur les articulations facettaires guidées par ultrasons sur des sujets humains entre le 1er novembre 1992 et le 1er novembre 2022. D’autres sources ont été tirées de listes de référence et de citations d’études pertinentes.

**Résultats:** Nous avons trouvé 48 études évaluant des interventions sur les articulations facettaires guidées par ultrasons. Le guidage par ultrasons pour l’injection des facettes cervicales et de leurs nerfs innervants avait une précision favorable (78 %-100 %) et une durée d’intervention inférieure à celle de la fluoroscopie ou de la tomodensitométrie, assorties d’un soulagement comparable de la douleur. La précision de l’injection intra-articulaire de la facette lombaire guidée par ultrasons (86 %-100 %) était plus fiable que celle du bloc de la branche médiane (72 %-97 %); l’analgésie était comparable au guidage fluoroscopique et tomodensitométrique. En général, ces procédures étaient plus difficiles pour les patients souffrant d’obésité, et les structures plus profondes étaient plus difficiles à cibler avec précision (par exemple, les niveaux cervicaux inférieurs, la branche dorsale L5).

**Conclusions:** Les interventions sur la facette articulaire guidées par ultrasons continuent d’évoluer. Certaines interventions techniquement difficiles peuvent être impraticables pour une utilisation généralisée ou nécessiter d’autres techniques. L’utilité du guidage par ultrasons en cas d’obésité et d’anatomie anormale peut être moindre.

## Introduction

Facet, or zygapophyseal, joints are a common cause of chronic spinal pain. Among patients with chronic spinal pain, the prevalence of facet joint–mediated pain is estimated to be 36% to 67% in the cervical spine,^1–4^ 42% to 48% in the thoracic spine,^3,5^ and 15% to 45% in the lumbar spine.^3,6–10^ Additionally, the prevalence of facet joint–mediated pain increases with age.^11^ As ubiquitous pain generators, facet joints are among the most common targets for therapeutic and diagnostic interventional techniques (i.e., intra-articular injection, medial branch block, and radiofrequency ablation), which may be used alongside physical therapy, self-management, and pharmacotherapy in the holistic management of chronic spinal pain.^1^

Indeed, local anesthetic injection of the nerves innervating the facet joints (i.e., medial branches) is a diagnostic standard for facet joint–mediated spinal pain.^12,13^ Facet joint intra-articular injection with local anesthetic also has some diagnostic utility, though it may be a relatively less reliable approach.^14^ The addition of a corticosteroid may prolong the analgesic effect from these interventions; however, this is not well supported in routine practice,^15–17^ particularly given concerns about negative systemic effects of repeated corticosteroid administration.^18^

A key benefit of establishing a diagnosis of facet joint–mediated spinal pain is that radiofrequency ablation may then be considered as a therapeutic intervention.^12,13^ Radiofrequency ablation, which uses thermal energy to coagulate the nerves innervating the offending facet joints, may provide longer duration analgesia than either facet joint intra-articular injection or medial branch block, without need for corticosteroid administration.

Although facet joint interventions have traditionally been performed with fluoroscopic guidance, the growing availability of ultrasound imaging has facilitated the development of new techniques for managing chronic spinal pain, potentially improving accessibility, safety, and effectiveness.^19^ Ultrasound guidance permits the avoidance of ionizing radiation exposure associated with traditional fluoroscopic or computed tomography (CT)-guided approaches and also facilitates real-time visualization of soft tissue and neurovascular structures around the site of intervention. The portability of ultrasound allows more interventions to be performed in the clinic setting, which is less resource intensive than the fluoroscopy suite. However, limitations of ultrasound guidance (e.g., image quality) present potential challenges during facet joint intervention.

This narrative review provides a broad overview of ultrasound-guided facet joint interventions. Its first objective is to provide a practical overview of facet joint and medial branch anatomy. The second objective is to present, based on the results of a systematic literature search, current techniques for ultrasound-guided facet joint interventions. This final objective is to synthesize data on the performance, safety, and efficacy of ultrasound-guided facet joint interventions.

## Methods

To provide a comprehensive review of this topic, the authors systematically searched the PubMed, MEDLINE, CINAHL, Embase, and Cochrane Central Register of Controlled Trials databases from November 1, 1992, to November 1, 2022. The search strategy was constructed with the assistance of a medical information specialist. The following MeSH (Medical Subject Headings) were used: “zygapophyseal joint” OR “facet joint” OR “vertebrae,” joined by the Boolean operator “AND” to the MeSH “interventional ultrasound” OR “ultrasonography” OR “injections” OR “pain management” OR “fluoroscopy” as well as “chronic pain” OR “back pain” OR “neck pain.” For our detailed search terms, refer to Supplement 1.

Inclusion criteria included studies of ultrasound-guided facet joint interventions (i.e., intra-articular injection, medial branch block, or radiofrequency ablation) involving human subjects (i.e., randomized controlled trials, case series, observational studies, anatomical studies, and cadaveric studies). Abstracts, book chapters, editorials, and reviews were excluded, but their reference lists were examined for relevant primary literature. There was no exclusion based on language. Article screening was performed by M.W., and M.R. was consulted in the event of ambiguity. Additional sources were drawn from the reference lists and citations of relevant studies.

In this review, the outcomes sought were divided into the following categories: (1) performance-related outcomes (i.e., accuracy of needle tip positioning, procedural duration, required number of attempts), (2) safety-related outcomes (i.e., adverse events, radiation exposure), and (3) efficacy-related outcomes (i.e., pain scores, participant rating of functional improvement).

The methodologic quality of included studies was also assessed. Case reports were evaluated using the CARE (CAse REport) checklist.^20^ Randomized controlled trials were assessed using the Cochrane Risk of Bias version 2 tool.^21^ Nonrandomized, prospective observational studies were assessed using the Risk of Bias in Non-Randomized Studies of Interventions tool, and retrospective observational studies were appraised using the Newcastle-Ottawa Scale.^22^ Cadaveric studies were appraised using the QUality Appraisal for Cadaveric Studies scale.^23^ Anatomic (i.e., radiographic) studies were assessed with the Anatomical Quality Assessment tool.^24^ This review was prepared in accordance with the Scale for the Assessment of Narrative Review Articles.^25^

## Results

**Figure 1.**
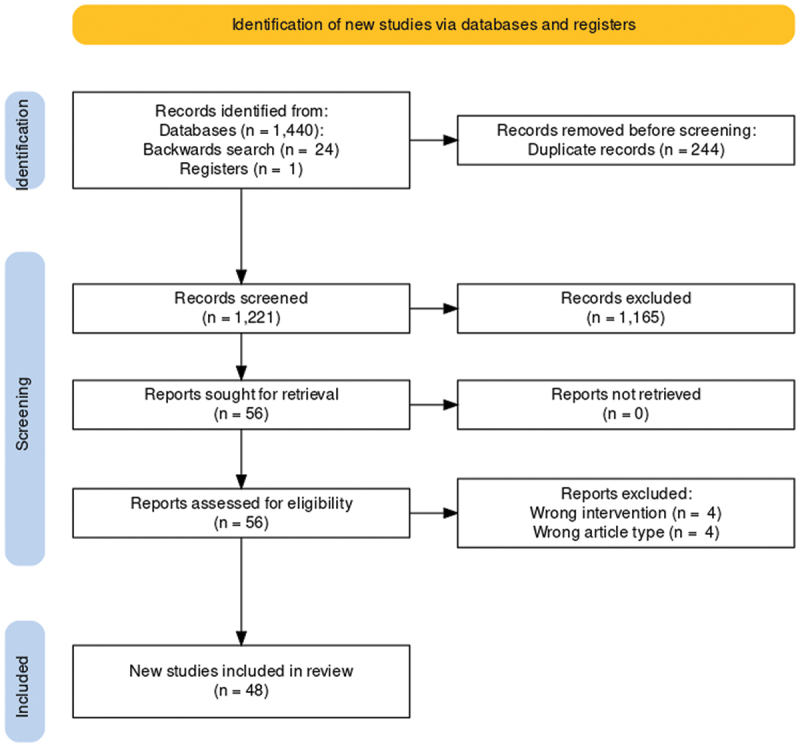
Summary of literature search.

The outcome of the literature search is summarized in Figure 1 and Table 1. We identified 48 studies that met our inclusion criteria, including 1741 human subjects in total, and over 3947 spinal levels were targeted. Among these subjects, there were 94 healthy volunteers, 1601 patients, and 46 cadavers. Nine of these studies were cadaveric studies, 2 were anatomic studies of living subjects, 3 were case reports, 16 were prospective observational studies, 7 were retrospective observational studies, 9 were randomized controlled trials, and 2 had mixed designs.^26,27^ The risk of bias assessment for the included studies is summarized in Table 2. In general, most of the included studies were at moderate risk of bias (For further details, refer to Supplemental Data).

**Table 1.** Summary of included studies from systematic literature search.

									Outcomes		
Study	Title	Study design	Subjects	Levels targeted	Region	Intervention	Comparator	Procedural	Safety	Efficacy	Comments
Finlayson et al.^43^	Real-time detection of periforaminal vessels in the cervical spine	Anatomic study	102 patients	1005	Cervical	—	—	—	Incidental periforaminal blood vessels were found at 24% of cervical levels surveyed	—	This sonoanatomical study assessed for incidental periforaminal blood vessels in patients scheduled for cervical medial branch block
Siegenthaler et al.^42^	Ultrasound anatomy of the nerves supplying the cervical zygapophyseal joints	Anatomic study	50 patients	50	Cervical	—	—	All cervical medial branches from C3 to C6 were detected in 78% of patients, but the C7 medial branch was only visualized in 32% of patients	—	—	This sonoanatomical study assessed for the visibility of cervical medial branches in patients with chronic neck pain
***Cervical facet joint intra-articular injection***											
Bodor et al.^44^	Ultrasound-guided cervical facet joint injections	Prospective, single-arm, observational study	36 patients	60	Cervical	Ultrasound-guided facet joint intra-articular injection	—	92% to 98% of injections showed intra-articular contrast spread, depending on criteria to confirm accurate placement	No complications noted	—	—
Freire et al.^45^	Ultrasound‐guided cervical facet joint injections	Cadaveric study	4 cadavers	40	Cervical	Ultrasound-guided facet joint intra-articular injection	—	Needle tip localization was satisfactory in 82% of injections, verified by demonstration of intra-articular or peri-articular latex dye on dissection	—	—	—
Galiano et al.^40^	Ultrasound-guided facet joint injections in the middle to lower cervical spine	Cadaveric study	4 cadavers	10	Cervical	Ultrasound-guided facet joint intra-articular injection	—	CT confirmation of needle tip localization was satisfactory in 100% of injections	—	—	Preclinical study to demonstrate feasibility of new technique
Obernauer et al.^46^	Ultrasound-guided versus computed tomography–controlled facet joint injections in the middle and lower cervical spine: A prospective randomized clinical trial	Randomized controlled trial	40 patients	54	Cervical	Ultrasound-guided facet joint intra-articular injection	CT-guided facet joint intra-articular injection	CT confirmation of needle tip localization was satisfactory in 100% of ultrasound-guided injections, without need for further needle repositioning. CT-guided injection required needle repositioning in 65% of cases. Ultrasound guidance required less time compared to CT guidance (5 min versus 11 min for single level; 6 min versus 15 min for two levels)	No complications were noted. Radiation exposure from CT-guided injection was 88 mGy·cm for single level and 205 mGy·cm for two-level intervention	Both groups had similar reduction in VAS scores at 1-month follow-up	—
***Cervical medial branch block***											
Eichenberger et al.^41^	Sonographic visualization and ultrasound-guided block of the third occipital nerve	Randomized controlled trial	14 healthy volunteers	28	Cervical	Ultrasound-guided third occipital nerve block	Sham ultrasound-guided third occipital nerve block (saline)	Fluoroscopic confirmation of needle tip localization was satisfactory in 82% of injections	No major complications were noted. There was one small subcutaneous hematoma	90% of third occipital nerve blocks produced anesthesia in the desired distribution, whereas no control blocks resulted in sensory changes	—
Finlayson et al.^50^	Cervical medial branch block: A novel technique using ultrasound guidance	Prospective, single-arm, observational study	53 patients	163	Cervical	Ultrasound-guided medial branch block	—	94% of injections were successful, as determined by appropriate contrast spread pattern. The median number of needle passes per level was 1.33	No complications were noted. Overlying blood vessels were incidentally seen in 11 patients	—	An additional 20 patients and 46 levels were injected in a preliminary phase of the study, with 100% satisfactory needle tip placement on fluoroscopy
Finlayson et al.^49^	A randomized comparison between ultrasound- and fluoroscopy-guided third occipital nerve block	Randomized controlled trial	40 patients	40	Cervical	Ultrasound-guided third occipital nerve block	Fluoroscopy-guided third occipital nerve block	Desired contrast spread pattern was comparable with ultrasound and fluoroscopic guidance (95% versus 100%). Procedure time and required number of needle passes were lower with ultrasound guidance compared to fluoroscopy (213 s versus 397 s; two versus six needle passes)	No complications were noted. In 10% of patients, incidental blood vessels were observed on ultrasound scan. There was a 10% rate of blood aspiration in the fluoroscopy group (0% with ultrasound), and no intravascular contrast spread in either group. With fluoroscopy, there was a 15% rate of C2–C3 intra-articular spread of contrast	Sensory testing and distribution of hypoesthesia were comparable in both groups	—
Finlayson et al.^48^	A prospective validation of biplanar ultrasound imaging for C5–C6 cervical medial branch blocks	Prospective, single-arm, observational study	40 patients	80	Cervical	Ultrasound-guided medial branch block	—	Fluoroscopic confirmation of needle tip localization was satisfactory in 99% of injections. The mean procedure time was 249 s	No complications were noted. Blood vessels were visualized in 21% of blocks	—	—
Finlayson et al.^33^	A randomized comparison between ultrasound- and fluoroscopy-guided C7 medial branch block	Randomized controlled trial	50 patients	50	Cervical	Ultrasound-guided medial branch block	Fluoroscopy-guided medial branch block	Success rate was similar for both ultrasound- and fluoroscopy-guided blocks (92% versus 96%). Compared to fluoroscopy, ultrasound guidance had a shorter procedure time (233 min versus 391 min) and required fewer needle passes (two versus four)	No complications were noted. In 40% of patients, ultrasound guidance revealed an overlying blood vessel, which was avoided. In several fluoroscopy-guided blocks, there was intravascular (20%) and intra-articular (4%) spread of contrast agent	After injection, both groups reported comparably decreased pain scores	—
Park et al.^54^	Spinal cord injury during ultrasound-guided C7 cervical medial branch block	Case report	1 patient	1	Cervical	Ultrasound-guided medial branch block	—	—	—	—	This case report describes a patient who underwent ultrasound-guided C7 medial branch block and had subsequent focal intramedullary hemorrhage at C7–T1, with residual upper limb weakness at 1-month follow-up
Park et al.^47^	Ultrasound versus fluoroscopy-guided cervical medial branch block for the treatment of chronic cervical facet joint pain: A retrospective comparative study	Retrospective, two-arm, observational study	126 patients	186	Cervical	Ultrasound-guided medial branch block	Fluoroscopy-guided medial branch block	Procedure time was lower with ultrasound guidance (221 s versus 383 s, and fewer needle passes were required (two versus five needle passes)	No major complications were noted. Both groups had a similar incidence of transient headache, vasovagal reaction, and pain exacerbation. Blood aspiration was 12% in with fluoroscopic guidance (0% with ultrasound). There were no instances of intravascular contrast spread	Both groups had comparable decreases in verbal numeric pain and Neck Disability Index scores at 6-month follow-up	—
Siegenthaler et al.^51^	Accuracy of ultrasound-guided nerve blocks of the cervical zygapophysial joints	Randomized controlled trial	60 healthy volunteers	180	Cervical	Ultrasound-guided medial branch block	—	On fluoroscopy, needle tip placement was accurate in 78% of cases, and the rate of desired contrast dye spread was 84%	No major complications were noted. Two volunteers reported transient neck pain	—	Of the 180 injections in this study, 73 were purposefully misplaced a priori to determine agreement statistics for two blinded raters examining fluoroscopy images
***Cervical medial branch radiofrequency ablation***											
Awad et al.^53^	Ultrasound-guided versus C-arm fluoroscopy controlled radiofrequency ablation of the cervical facets	Prospective, two-arm, observational study	40 patients	123	Cervical	Ultrasound-guided medial branch radiofrequency ablation	Fluoroscopy-guided medial branch radiofrequency ablation	Procedural time was lower with ultrasound guidance (10 min versus 14 min)	No major complications were noted. Both groups had a similar incidence of transient pain aggravation, paresthesia, allergic reaction, and superficial infection. One patient had mild upper limb weakness that resolved by 1-month follow-up (technique unspecified)	Both groups had comparable decreases in VAS scores at 1-month follow-up	—
Kim et al.^39^	Ultrasound-guided pulsed radiofrequency of the third occipital nerve	Case report	2 patients	2	Cervical	Ultrasound-guided pulsed radiofrequency ablation of third occipital nerve	NA	Needle tip placement was confirmed by fluoroscopy in both patients	No complications were noted	VAS scores were persistently reduced up to 4-month and 12-month follow-up in the two patients, respectively	—
Lee et al.^52^	Ultrasound-guided radiofrequency neurotomy in cervical spine: Sonoanatomic study of a new technique in cadavers	Cadaveric study	5 cadavers	34	Cervical	Ultrasound-guided medial branch radiofrequency ablation	—	87% of radiofrequency ablations were successful, as determined by histologic examination of the medial branches	—	—	—
Stulc et al.^55^	Ultrasound‐guided thoracic facet injections	Cadaveric study	1 cadaver	20	Thoracic	Ultrasound-guided facet joint intra-articular injection	—	80% of injections showed intra-articular contrast spread	—	—	—
***Lumbar facet joint intra-articular injection***											
Galiano et al.^57^	Ultrasound guidance for facet joint injections in the lumbar spine: A CT-controlled feasibility study	Cadaveric study	5 cadavers	10	Lumbar	Ultrasound-guided facet joint intra-articular injection	—	There was 100% accuracy of needle tip placement, as confirmed by CT	—	—	Only one cadaver underwent needle placement; remaining four were used for studying sonoanatomy
Galiano et al.^58^	Ultrasound-guided versus computed tomography–controlled facet joint injections in the lumbar spine: A prospective randomized clinical trial	Randomized controlled trial	40 patients	40	Lumbar	Ultrasound-guided facet joint intra-articular injection	CT-guided facet joint intra-articular injection	CT assessment confirmed accurate needle tip placement in 94% of all ultrasound-guided injections. There was reduced time to needle placement with ultrasound-guidance compared to CT guidance (14 min versus 22 min)	No major complications were noted. One patient reportedly had fluid retention and peripheral edema, possibly but not clearly related to corticosteroid administration. The mean radiation dose for CT guidance was 364 mGy·cm	Both ultrasound-guided and CT-guided injections resulted in similar decreases in VAS Scores at 6-week follow-up	—
Gofeld et al.^63^	Ultrasound-guided injection of lumbar zygapophyseal joints	Cadaveric study	5 cadavers	50	Lumbar	Ultrasound-guided facet joint intra-articular injection	—	The success rate for needle-tip placement was 88%, as determined by fluoroscopy and contrast dye spread pattern	—	—	—
Ha et al.^80^	Comparison of ultrasonography- and fluoroscopy-guided facet joint block in the lumbar spine	Retrospective, two-arm, observational study	105 patients	105	Lumbar	Ultrasound-guided facet joint intra-articular injection	Fluoroscopy-guided facet joint intra-articular injection	—	There was a similar incidence of transient, minor adverse effects with both ultrasound- and fluoroscopy-guided injections	VAS and ODI scores were comparably reduced between ultrasound- and fluoroscopy-guided injections at 6-week follow-up. Procedure time was comparable between ultrasound- and fluoroscopy-guided injections. Patients were billed approximately 50% more for fluoroscopy-guided injections than ultrasound-guided injections	—
Karkucak et al.^81^	Comparison of clinical outcomes of ultrasonography-guided and blind local injections in facet syndrome: A 6-week randomized controlled trial	Randomized controlled trial	47 patients	—	Lumbar	Ultrasound-guided facet joint intra-articular injection	Landmark-guided facet joint intra-articular injection	—	—	Ultrasound guidance yielded a greater decrease in VAS scores at 6-week follow-up, whereas ODI decreased to a similar degree in both groups	—
Massone et al.^67^	Real-time fusion imaging in low back pain: A new navigation system for facet joint injections	Retrospective, two-arm, observational study	65 patients	183	Lumbar	Ultrasound fusion imaging-guided facet joint intra-articular injection	CT-guided facet joint intra-articular injection	Procedural time was comparable between fusion imaging– and CT-guided injections (21 min for both)	No major complications were noted. Several patients in each group had a mild subcutaneous hematoma	VAS and ODI scores were comparably decreased in both groups at 2-month follow-up. Patient satisfaction was comparable between both groups	The fusion imaging–guided technique involved real-time registration of sonographic imaging against prior CT or magnetic resonance imaging, with magnetic needle tip tracking
Rasoulian et al.^82^	Ultrasound-guided spinal injections: A feasibility study of a guidance system	Prospective, single-arm, observational study	4 patients	5	Lumbar	Ultrasound fusion imaging-guided facet joint intra-articular injection	—	—	—	—	Proof of concept study to demonstrate high degree of precision for fusion image guidance and needle tip tracking. Clinical outcomes not reported
Sadeghian and Motiei-Langroudi^78^	Sonography guided lumbar nerve and facet blocks: The first report of clinical outcome from Iran	Prospective, single-arm, observational study	14 patients	18	Lumbar	Ultrasound-guided facet joint intra-articular injection or medial branch block	—	—	No complications were noted.	VAS scores were decreased at 1-week follow-up	This was a mixed population. Four patients received selective nerve root block and ten patients received facet joint intra-articular injection
Santiago^89^	Ultrasound-guided facet block to low back pain: A case report	Case report	1 patient	3	Lumbar	Ultrasound-guided facet joint intra-articular injection	—	—	No complications noted	Numeric pain scores were decreased at 5-month follow-up	—
Sartoris et al.^65^	In vivo feasibility of real-time MR–US fusion imaging lumbar facet joint injections	Prospective, single-arm, observational study	38 patients	112	Lumbar	Ultrasound fusion imaging–guided facet joint intra-articular injection	—	Needle tip placement with ultrasound and magnetic positioning system guidance yielded 86% accuracy as assessed with fluoroscopy. The mean procedural time was 28 min	No major complications were noted. Ten patients had transient, mild subcutaneous hematoma at the injection site	VAS scores were decreased at 8-week follow-up	—
Tay et al.^74^	Ultrasound-guided lumbar spine injection for axial and radicular pain: A single institution early experience	Retrospective, single-arm, observational study	27 patients	—	Lumbar	Ultrasound-guided facet joint intra-articular injection	—	—	No complications noted	Reduced numeric rating scale and ODI scores at 3-month follow-up	This was a mixed population with some patients also receiving selective nerve root injections in addition to facet joint intra-articular injection and additional patients receiving selective nerve root injections only. The aggregate results of the entire sample were presented
Wen et al.^64^	[A clinical trial of ultrasound-guided facet joint block in the lumbar spine to treat facet joint related low back pain]	Randomized controlled trial	20 patients	35	Lumbar	Ultrasound-guided facet joint intra-articular injection	Landmark-guided facet joint intra-articular injection	CT confirmation of needle tip localization was satisfactory in 86% of ultrasound-guided and 31% of landmark-guided injections. Procedural time was lower with ultrasound guidance compared to landmark technique (206 s versus 397 s)	—	VAS scores were decreased in both groups at 6-week follow-up. Although ultrasound-guided injection resulted in lower VAS scores compared to landmark technique, at 30 min there was no significant difference at any other time point	—
Ye et al.^26^	Ultrasound-guided versus low dose computed tomography scanning guidance for lumbar facet joint injections: Same accuracy and efficiency	Mixed methods (anatomic study; randomized controlled trial)	50 patients	74	Lumbar	Ultrasound-guided facet joint intra-articular injection	CT-guided facet joint intra-articular injection	Needle tip positioning was accurate in 86% of ultrasound-guided facet joint intra-articular injections, as assessed with CT assessment	No major complications were noted. A comparable number of patients in both groups had a transient aggravation of low back pain	At 6-week follow-up, VAS scores were comparably improved in both groups and a similar proportion of patients still reported at least 50% pain reduction	Ten of the 40 patients did not receive injections and participated solely for assessment of sonoanatomy
Yun et al.^66^	Efficacy of ultrasonography-guided injections in patients with facet syndrome of the low lumbar spine	Randomized controlled trial	57 patients	185	Lumbar	Ultrasound-guided facet joint intra-articular injection	Fluoroscopy-guided facet joint intra-articular injection	Ultrasound-guidance had slightly longer procedural time compared to fluoroscopy (263 s versus 249 s)	No major complications were noted.	Comparable improvements in VAS, ODI, physician’s global assessment, and patient’s global assessment scores at 3-month follow-up	—
***Lumbar medial branch block***											
Erdogan et al.^62^	Accuracy of the anatomic placement in ultrasonography guided facet joint blockage with supervising of C-arm fluoroscopy	Prospective, single-arm, observational study	22 patients	67	Lumbar	Ultrasound-guided medial branch block	—	There was an appropriate contrast spread pattern in 91% of injections	No complications were noted	VAS scores were decreased postprocedurally, with variable follow-up	—
Etheridge et al.^60^	Ultrasound-guided L5 dorsal ramus block: Validation of a novel technique	Prospective, single-arm, observational study	100 patients	100	Lumbar	Ultrasound-guided L5 dorsal ramus injection block	—	Fluoroscopic confirmation of needle tip localization was satisfactory in 97% of injections but appropriate contrast spread was only seen in 95% of injections, indicating possible intravascular injection	One patient reported a small hematoma	—	—
Greher et al.^28^	Ultrasound-guided lumbar facet nerve block	Cadaveric study	5 cadavers	50	Lumbar	Ultrasound-guided medial branch block	—	The rate of successful needle tip placement at the desired radiographic endpoint was 90%, though contrast spread to the target site was observed in 94% of injections	—	—	—
Greher et al.^27^	Ultrasound-guided lumbar facet nerve block	Mixed methods (cadaveric study; prospective, single-arm, observational study)	1 cadaver, 20 healthy volunteers, 5 patients	28	Lumbar	Ultrasound-guided medial branch block	–	The success rate for needle tip placement was 89%, as determined by fluoroscopy. Procedure time to complete four to six injections was at most 40 min	No major complications	40% of patients were pain free 30 min postinjection and remaining patients reported 50% reduction in pain	Cadaver was used to develop ultrasound-guided injection technique. Healthy volunteers contributed to characterization of sonoanatomy but were not injected
Greher et al.^59^	Ultrasound-guided approach for L5 dorsal ramus block and fluoroscopic evaluation in unpreselected cadavers	Cadaveric study	10 cadavers	20	Lumbar	Ultrasound-guided L5 dorsal ramus injection block	—	Fluoroscopic confirmation of needle tip localization was satisfactory in 80% of injections	—	—	—
Han et al.^73^	Ultrasound versus fluoroscopy-guided medial branch block for the treatment of lower lumbar facet joint pain	Retrospective, two-arm, observational study	146 patients	—	Lumbar	Ultrasound-guided medial branch block	Fluoroscopy-guided medial branch block	Procedure time was lower with ultrasound guidance compared to fluoroscopic guidance (323 s versus 430 s)	No major complications were noted. Rates of transient headaches, vasovagal reactions, and low back pain aggravation were comparable with both ultrasound and fluoroscopic guidance. Blood aspiration occurred with fluoroscopic guidance (7%) but was not observed with ultrasound guidance	Verbal numeric pain scale and ODI scores decreased similarly with ultrasound and fluoroscopic guidance at 6-month follow-up	—
Hashemi et al.^71^	Ultrasound guidance for interventional pain management of lumbar facet joint pain: An anatomical and clinical study	Prospective, single-arm, observational study	30 patients	89	Lumbar	Ultrasound-guided medial branch block	—	There was 98% accuracy of needle tip placement as confirmed with fluoroscopy	No complications were noted	Verbal numeric pain scale and ODI scores decreased similarly with ultrasound and fluoroscopic guidance at 6-month follow-up	—
Jung et al.^70^	The validation of ultrasound-guided lumbar facet nerve blocks as confirmed by fluoroscopy	Prospective, single-arm, observational study	50 patients	95	Lumbar	Ultrasound-guided medial branch block	—	There was a desired contrast spread pattern in 92% of medial branch blocks	—	Visual analog scores were decreased at 3-day follow-up	—
Putzu and Marchesini^76^	Ultrasound block of the medial branch: Learning the technique using CUSUM curves	Prospective, single-arm, observational study	14 patients	40	Lumbar	Ultrasound-guided medial branch block	—	Needle-tip placement was accurate in 72% of cases, as confirmed with fluoroscopy	—	—	This study analyzed the learning curve for experienced regional anesthesiologists to acquire proficiency in ultrasound-guided lumbar medial branch block. Patient outcomes were not reported
Rauch et al.^68^	Ultrasound-guided lumbar medial branch block in obese patients	Prospective, single-arm, observational study	20 patients	84	Lumbar	Ultrasound-guided medial branch block	—	The success rate for needle-tip placement was 62%, as determined by fluoroscopy. Average procedural time was 5 min	—	Verbal rating scales were decreased at 24-h follow-up	—
Shim et al.^72^	Ultrasound-guided lumbar medial-branch block: A clinical study with fluoroscopy control	Prospective, single-arm, observational study	20 patients	101	Lumbar	Ultrasound-guided medial branch block	CT-guided medial branch block	Ultrasound-guided needle tip placement was accurate in 95% of cases, as confirmed with fluoroscopy; however, two injections had intravascular spread of contrast dye	No complications were noted	VAS scores were decreased immediately after the injections, comparable to CT-guided injection	—
Soni et al.^69^	Diagnostic ultrasound‐guided lumbar medial branch block of dorsal ramus in facet joint arthropathy: Technical feasibility and validation by fluoroscopy	Prospective, single-arm, observational study	60 patients	161	Lumbar	Ultrasound-guided medial branch block	—	The success rate was 86%, as determined by fluoroscopic verification of needle tip placement and contrast spread pattern	No complications were noted	Numeric rating scale scores were decreased at 24 h postprocedure, with 75% of patients reporting a decrease in numeric rating scale score of at least 50%	—
***Lumbar medial branch radiofrequency ablation***											
Gofeld et al.^61^	Magnetic positioning system and ultrasound guidance for lumbar zygapophysial radiofrequency neurotomy	Cadaveric study	6 cadavers	60	Lumbar	Ultrasound- and magnetic positioning system–guided medial branch radiofrequency ablation	Fluoroscopy-guided medial branch radiofrequency ablation	Needle tip placement with ultrasound and magnetic positioning system guidance yielded 97% accuracy as assessed with fluoroscopy. Procedure time for ultrasound and magnetic positioning system guidance was comparable to fluoroscopy guidance	—	—	—
***Lumbar medial branch cryoneurolysis***											
Kastler et al.^38^	Lumbar medial branch cryoneurolysis under ultrasound guidance: Initial report of five cases	Prospective, single-arm, observational study	5 patients	8	Lumbar	Ultrasound-guided medial branch cryoneurolysis	—	There was 100% accuracy of needle tip placement as confirmed with fluoroscopy	No complications noted	VAS and ODI scores were decreased at 3-month follow-up. Mean self-reported improvement was 77% at 12-month follow-up. There was one patient who did not benefit from the procedure	—

CUSUM = cumulative sum; ODI = Oswestry Disability Index; VAS = visual analogue scale.

**Table 2.** Risk of bias assessment of included studies.

Study	Study design	CARE	RoB2	QUACS	NOS	ROBINS-I	AQUA
Awad et al.^53^	Prospective, two-arm, observational study	—	—	—	—	Moderate risk	—
Bodor et al.^44^	Prospective, single-arm, observational study	—	—	—	—	Low risk	—
Çırak and Okur^79^	Retrospective, single-arm, observational study	—	—	—	7	—	—
Eichenberger et al.^41^	Randomized controlled trial	—	Low risk	—	—	—	—
Erdogan et al.^62^	Prospective, single-arm, observational study	—	—	—	—	Moderate risk	—
Etheridge et al.^60^	Prospective, single-arm, observational study	—	—	—	—	Moderate risk	—
Finlayson et al.^43^	Anatomic study	—	—	—	—	—	Low risk
Finlayson et al.^48^	Prospective, single-arm, observational study	—	—	—	—	Moderate risk	—
Finlayson et al.^50^	Prospective, single-arm, observational study	—	—	—	—	Low risk	—
Finlayson et al.^33^	Randomized controlled trial	—	Some concerns	—	—	—	—
Finlayson et al.^49^	Randomized controlled trial	—	Low risk	—	—	—	—
Freire et al.^45^	Cadaveric study	—	—	11	—	—	—
Galiano et al.^40^	Cadaveric study	—	—	8	—	—	—
Galiano et al.^57^	Cadaveric study	—	—	7	—	—	—
Galiano et al.^58^	Randomized controlled trial	—	Some concerns	—	—	—	—
Gofeld et al.^63^	Cadaveric study	—	—	8	—	—	—
Gofeld et al.^61^	Cadaveric study	—	—	9	—	—	—
Greher et al.^59^	Cadaveric study	—	—	13	—	—	—
Greher et al.^27^	Cadaveric study	—	—	11	—	—	—
Greher et al.	Mixed methods (cadaveric study; prospective, single-arm, observational study)	—	—	8	—	Low risk	—
Ha et al.^80^	Retrospective, two-arm, observational study	—	—	—	6	—	—
Han et al.^73^	Retrospective, two-arm, observational study	—	—	—	6	—	—
Hashemi et al.^71^	Prospective, single-arm, observational study	—	—	—	—	Serious risk	—
Jung et al.^70^	Prospective, single-arm, observational study	—	—	—	—	Moderate risk	—
Karkucak et al.^81^	Randomized controlled trial	—	Some concerns	—	—	—	—
Kastler et al.^38^	Prospective, single-arm, observational study	—	—	—	—	Moderate risk	—
Kim et al.^39^	Case report	Moderate risk	—	—	—	—	—
Lee et al.^58^	Cadaveric study	—	—	10	—	—	—
Massone et al.^67^	Retrospective, two-arm, observational study	—	—	—	7	—	—
Obernauer et al.^46^	Randomized controlled trial	—	Some concerns	—	—	—	—
Park et al.	Case report	Moderate risk	—	—	—	—	—
Park et al.	Retrospective, two-arm, observational study	—	—	—	5	—	—
Putzu and Marchesini^76^	Prospective, single-arm, observational study	—	—	—	—	Low risk	—
Rasoulian et al.^82^	Prospective, single-arm, observational study	—	—	—	—	Low risk	—
Rauch et al.^68^	Prospective, single-arm, observational study	—	—	—	—	Moderate risk	—
Sadeghian and Motiei-Langroudi^78^	Prospective, single-arm, observational study	—	—	—	—	Moderate risk	—
Santiago^89^	Case report	Moderate risk	—	—	—	—	—
Sartoris et al.^65^	Prospective, single-arm, observational study	—	—	—	—	Moderate risk	—
Shim et al.^72^	Prospective, single-arm, observational study	—	—	—	—	Serious risk	—
Siegenthaler et al.^42^	Anatomic study	—	—	—	—	—	High risk
Siegenthaler et al.^51^	Randomized controlled trial	—	Some concerns	—	—	—	—
Soni et al.^69^	Prospective, single-arm, observational study	—	—	—	—	Moderate risk	—
Stulc et al.^55^	Cadaveric study	—	—	9	—	—	—
Tay et al.^74^	Retrospective, single-arm, observational study	—	—	—	7	—	—
Touboul et al.^77^	Retrospective, two-arm, observational study	—	—	—	5	—	—
Wen et al.^64^	Randomized controlled trial	—	Some concerns	—	—	—	—
Ye et al.^26^	Mixed methods (anatomic study; randomized controlled trial)	—	Some concerns	—	—	—	High risk
Yun et al.^66^	Randomized controlled trial	—	Some concerns	—	—	—	—

AQUA = Anatomical Quality Assessment tool; CARE = CAse REport checklist; NOS = Newcastle–Ottawa Scale (maximum score 9); QUACS = QUality Appraisal for Cadaveric Studies scale (maximum score 13); RoB2 = Cochrane Risk of Bias version 2 tool; ROBINS-I = Risk of Bias in Non-Randomized Studies of Interventions tool.

Data from the included studies are organized according to spinal region (i.e., cervical, thoracic, and lumbar), with subsections describing the performance of facet joint intra-articular injections and medial branch blocks, followed by a synthesis of the available evidence. The limitations of ultrasound guidance for performing spinal facet interventions are also reviewed, and practical recommendations are provided.

## Facet Joint Anatomy

Facet joints are paired joints formed by the superior articular process of one vertebra and the inferior articular process of the level above.^29^ The articular facets of these joints are covered with hyaline cartilage and enclosed in a synovial capsule, with the total volume of each facet joint being approximately 1 mL. Facet joints provide axial stability and define the spine’s range of motion at each region. Facet joint degenerative changes may involve bony hypertrophy, loss of cartilage and synovial fluid, and associated inflammation, which may all drive spinal pain.^30^ However, incidental and asymptomatic facet joint degeneration is also common,^31^ and joint capsular disruption may also cause pain in the absence of obvious radiographic changes.^32^

**Figure 2.**
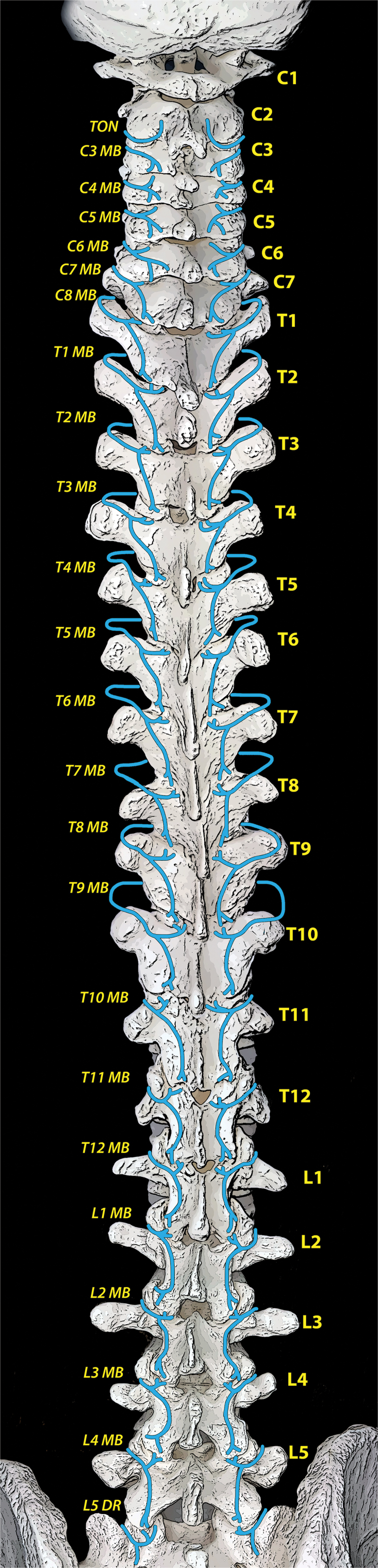
Facet joint anatomy and localization of their innervating nerves. DR = dorsal root; MB = medial branch.

Facet joint sensory innervation is provided by the medial branches, which are terminal divisions of each nerve root’s dorsal ramus (Figure 2).^33–36^ In general, each facet joint receives dual innervation, from the same level and also the level above (e.g., the C3–C4 facet joint is innervated by the C3 and C4 medial branches, and the L2–L3 facet joint is innervated by the L1 and L2 medial branches); therefore, both contributing medial branches must be targeted to block sensation for a given facet joint. The localization of the medial branches varies depending on the region of the spine.^29,37,38^ Most cervical medial branches are found on the lateral waist of their respective vertebrae’s articular pillars. However, the superficial medial branch of C3 (third occipital nerve; TON) crosses the lateral surface of the C2–C3 joint, which it innervates, and the C7 medial branch is found at the junction of the C7 superior articular process and transverse process. Medial branches arising from T1 to T4 and T9 to T10 cross the superolateral margins of the transverse process below and then continue inferomedially. From T5 to T8, the medial branches are suspended just superior to the transverse process, in the intertransverse space. The T11 and T12 medial branches follow a course similar to that of the lumbar medial branches, which reliably pass over the junction of the transverse process and superior articular process of the level below. The L5–S1 facet joint is unique in that its innervation is thought to arise from the L5 dorsal ramus itself, rather than a discrete medial branch.^29^

## Ultrasound-Guided Interventions

The most common procedures performed on facet joints include intra-articular injections, blocks of the nerves innervating the joints, and their denervation using radiofrequency ablation. Cryoablation and pulsed radiofrequency ablation of these structures are also described, albeit rarely.^39,40^ Though facet joint intra-articular injection has clinically been used for both diagnostic and therapeutic purposes, the gold standard for diagnosing facet joint pain is medial branch block, typically using local anesthetic volumes of 0.5 mL or less.^12,13^ Usually, a high-frequency linear ultrasound transducer is used for superficial structures (i.e., cervical or thoracic facet joints), whereas a low-frequency curvilinear transducer is better suited for deeper targets (i.e., lumbar facet joints).

### Cervical Facet Interventions

#### Cervical Facet Joint Intra-Articular Injection

**Figure 3.**
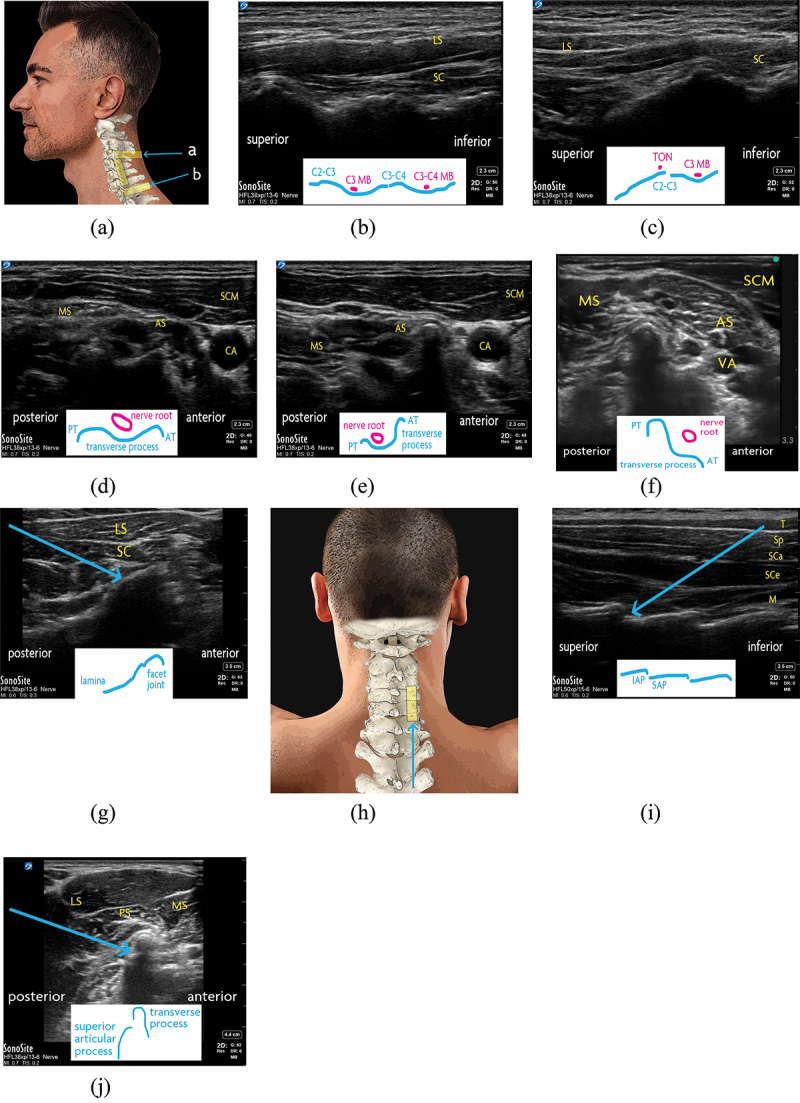
Cervical facet joint interventions. (a) Surface anatomy of the lateral neck. The ultrasound transducer positions for TON and C7 facet injections, as well as coronal scan, are demonstrated with yellow rectangles. In-plane needle trajectories for (a) TON block and (b) C7 facet joint intra-articular injection are shown with blue arrows. (b) Cervical facet joints and medial branches, as seen with an ultrasound transducer in coronal orientation on the lateral neck. A distinct “sawtooth” pattern is demonstrated, with the facet joints seen as peaks and the articular pillars appearing as troughs. (c) C2–C3 facet joint, as seen with an ultrasound transducer in coronal orientation on the lateral neck. Superior to the joint is a characteristic “drop-off.” The third occipital nere is shown atop the C2–C3 facet joint. (d) The C5 transverse process, as seen with an ultrasound transducer in transverse orientation on the lateral neck. The anterior and posterior tubercles of the C5 transverse process are of comparable size. (e) The C6 transverse process, as seen with an ultrasound transducer in transverse orientation on the lateral neck. The defining feature of the C6 vertebra is its large anterior tubercle (Chassaignac’s tubercle). (f) The C7 transverse process, as seen with an ultrasound transducer in transverse orientation on the lateral neck. The posterior tubercle of C7 is more prominent than the diminuitive anterior tubercle. (g) Cervical facet intra-articular injection, as seen with an ultrasound transducer in transverse position on the lateral neck. The needle (blue arrow) is advanced in-plane to the target from a posterior to anterior trajectory. By translating the transducer superiorly or inferiorly to focus on the articular pillar, the respective medial branch may be targeted in similar fashion. (h) Surface anatomy of the posterior neck. The ultrasound transducer position for cervical facet intra-articular injection is demonstrated with a yellow rectangle, and the in-plane needle trajectory is shown with a blue arrow. (i) Cervical facet joints, as seen with an ultrasound transducer in saggital orientation on the posterior neck. For intra-articular injection, the needle is advanced in-plane to the target from an inferior-to-superior trajectory (blue arrow). (j) C7 medial branch block, as seen with an ultrasound transducer in coronal position on the lateral neck. The needle is advanced in-plane to the junction of the superior articular process and transverse process from a posterior-to-anterior trajectory (blue arrow). AS = anterior scalene muscle; AT = anterior tubercle; CA = carotid artery; IAP = inferior articular process; LS = levator scapulae muscle; M = multifidus muscle; MB = medial branch; MS = middle scalene muscle; PS = posterior scalene muscle; PT = posterior tubercle; SAP = superior articular process; SC = semispinalis capitis; SCa = semispinalis capitis muscle; SCe = semispinalis cervicis muscle; SCM = sternocleidomastoid muscle; Sp = splenius muscle; T = trapezius muscle; VA = vertebral artery.

Ultrasound-guided cervical facet joint intra-articular injection was initially described by Galiano and colleagues with the patient lying in decubitus (Figure 3a).^41^ With the transducer oriented coronally on the lateral neck, the facet column appears as a characteristic wavy hyperechoic line (“sawtooth pattern”; Figure 3b), and the articular pillars appear as troughs and the facet joints as peaks.

Several characteristic structures can be used to help identify the target level. The C2 inferior articular process has a distinctive drop-off before the C1 transverse process appears slightly superior to it (Figure 3c). The C2–C3 facet joint is also inferior and slightly anterior to the mastoid process, and anterosuperior to that is the vertebral artery. The levels may also be identified with the transducer placed transversely in midline to view the spinous processes. C1 is immediately caudal to the occiput and has, at most, a rudimentary spinous process, whereas C2 has a prominent bifid spinous process. Inferiorly, the vertebral levels can be verified by viewing the transverse processes of C5, C6, and C7 (Figure 3d–f). The C7 transverse process has a rudimentary anterior tubercle, the C6 transverse process has a very prominent anterior tubercle, and the C5 transverse process has more equally sized tubercles.

With the target facet joint visualized, the transducer is typically rotated transversely to allow in-plane needle placement with a posterior-to-anterior trajectory (Figure 3a,g). Alternatively, a posterior approach may be considered with the patient positioned prone (Figure 3h,i), which allows bilateral injections to be performed without repositioning the patient. At the target level, an in-plane inferior-to-superior needle trajectory is used.

#### Cervical Medial Branch Block

Eichenberger et al. introduced the ultrasound-guided technique for blocking the TON.^42^ The patient is positioned lateral decubitus. The lateral aspect of the neck is first scanned at the level of the mastoid, with the transducer in a coronal plane. The TON itself may be visible at the level of the C2–C3 joint, which is localized as described above. However, to perform the block, TON visualization is not strictly necessary. After identifying the C2–C3 joint space, the transducer is rotated transversely, and the needle is inserted in-plane from a posterolateral entry point until periosteum is contacted. If desired, the needle tip position may be confirmed before injection by rotating the transducer coronally again to demonstrate the needle tip by the TON and C2–C3 facet.

From C3 to C6, the medial branches are targeted at the center of their respective articular pillars, seen with the transducer oriented coronally on the lateral neck (Figure 3b). The medial branches may themselves be visible on the articular pillars. At the desired level, the transducer is rotated transversely and the needle is advanced in-plane from a posterolateral entry point until the articular pillar is contacted (Figure 3g). Note that the cervical articular processes are located posterior to the posterior tubercules. The lower levels of C5 and C6 are technically challenging due to increased target depth in the base of the neck; therefore, it may be especially helpful to dynamically scan the anatomy and verify needle tip placement with biplanar imaging.

For TON and C3 to C6 medial branch blocks alike, an alternative approach has also been described, where the target is identified from the coronal transducer orientation and injected with an out-of-plane approach, without rotating the transducer to a transverse position for an in-plane trajectory.

The C7 medial branch requires a different technique for intervention due to the unique anatomy of the C7 vertebra.^34^ Ultrasound transducer positioning may also be impeded by the clavicle.^43^ The C7 and T1 transverse processes are first located by scanning with the transducer transversely oriented at the lower part of the lateral neck. The target is the C7 superior articular process, immediately posterior to the C7 transverse process (Figure 3j). Alternatively, if the C7 superior articular process is not apparent, the part of the C7 transverse process immediately caudal to the C6–C7 facet joint is targeted. For either target, an in-plane needle trajectory is used with a posterolateral insertion site. Needle tip placement may be confirmed by scanning with the transducer in a coronal orientation.

For facet joint interventions at the cervical level, care must be taken to maintain strict control of the needle tip during manipulation. The cervical nerve roots are in close proximity to the cervical facet joints, as are numerous important vascular structures (i.e., vertebral artery, superficial and deep cervical arteries, inferior thyroid artery). Thorough scanning of the local sonoanatomy may be helpful for procedural planning given a high incidence of blood vessels overlying structures of interest for cervical facet joint interventions.^44^

#### Evidence for Cervical Facet Interventions

Seventeen of the included studies were focused on cervical facet joint interventions, of which five were observational studies and five were randomized controlled trials (Table 1). Ten studies examined interventions targeting the nerves innervating the facet joints (i.e., TON, medial branches), and five studies assessed facet joint intra-articular injection. Notably, there were two studies of ultrasound-guided cervical medial branch radiofrequency ablation, one in cadavers and another in patients.

##### Performance-Related Outcomes

The accuracy of ultrasound-guided cervical facet joint intra-articular injection ranges from 78% to 100%,^41,45–47^ with a potentially higher failure rate at the more challenging lower levels.^46^ One randomized trial compared ultrasound-guided facet joint intra-articular injection with CT guidance in 40 patients with facet-mediated mid- to low-cervical spine pain and reported that ultrasound guidance had superior accuracy on first attempt (100% versus 35%) and had a shorter procedural time (6 min versus 14 min).^47^ Similarly, a retrospective observational study found that ultrasound guidance for medial branch block required less procedural time (221 s versus 383 s) and fewer needle passes (two versus five), compared to fluoroscopy.^48^

Finlayson and colleagues conducted a series of comparative studies examining ultrasound-guided local anesthetic injection of nerves innervating the cervical facet joints; they reported comparable accuracy to fluoroscopy-guided injection (95%–100%), as determined by fluoroscopic confirmation of needle tip position.^34,49–51^ Other investigators had somewhat lower accuracy with medial branch and TON injection, according to fluoroscopic confirmation (78%–82%).^42,46,52^

In a cadaver study, Lee and colleagues reported 100% successful ultrasound-guided radiofrequency cannula placement as verified by fluoroscopy, and dissection revealed successful ablation in 30 of 34 medial branches targeted; C6 and C7 were the only levels where medial branches were unsuccessfully coagulated.^53^ In an observational study, ultrasound guidance was found to have a lower procedural time for radiofrequency ablation compared to CT guidance (10 min versus 14 min).^54^

##### Safety

There was one case report of spinal cord injury following ultrasound-guided C7 medial branch block, with persistent neurologic deficits after 1 month.^55^ Otherwise, there were no major complications observed in any other clinical studies. Transient minor adverse effects were infrequently observed (e.g., vasovagal reaction, pain exacerbation). In an anatomic study of 102 patients with chronic neck pain, 24% of cervical levels were found to have incidental blood vessels in the vicinity of the cervical medial branches.^44^ Some studies reported a 10% to 20% rate of blood aspiration with fluoroscopy-guided medial branch or TON block, though this did not result in patient morbidity.^34,48,50^ Blood aspiration was not noted with ultrasound guidance in any study.

##### Efficacy

In observational studies and randomized controlled trials of ultrasound-guided injection of cervical facet joints and the nerves that innervate them, ultrasound guidance produced comparable reductions in pain and disability scores when compared with fluoroscopy or CT guidance.^40,42,47–50,54^ In a retrospective study of 126 patients, ultrasound- and fluoroscopy-guided cervical medial branch injection with corticosteroid produced comparable reductions in pain severity and disability, lasting at least 6 months.^48^

In the only study comparing ultrasound- and fluoroscopy-guided cervical radiofrequency ablation,^53^ Awad et al. found comparable analgesia at one month follow-up.^54^ Pulsed radiofrequency ablation of the TON has also been reported, with analgesic benefit up to 12 months.^40^

### Thoracic Facet Interventions

#### Thoracic Facet Joint Intra-Articular Injection

**Figure 4.**
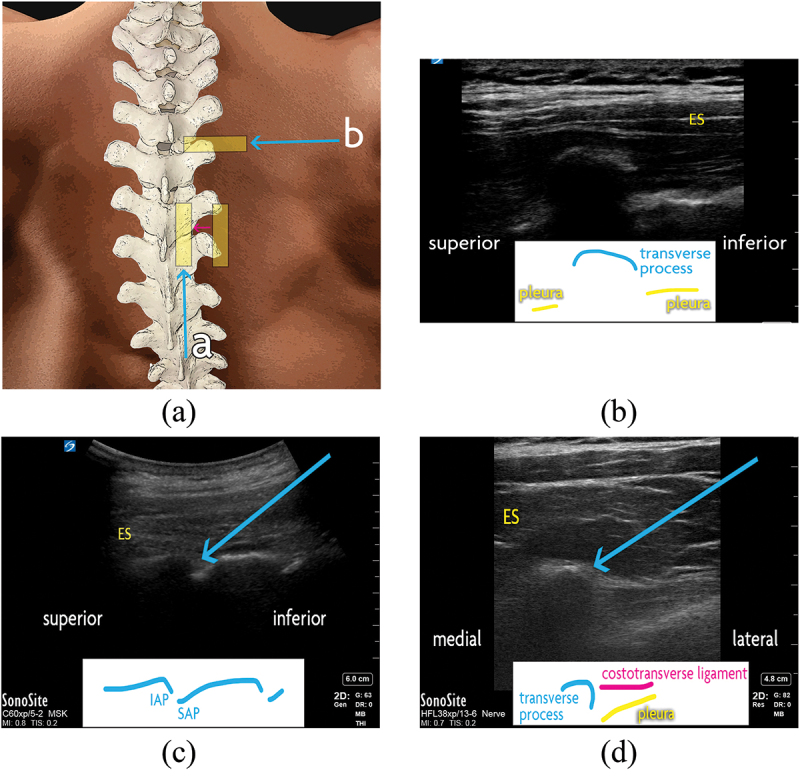
Thoracic facet joint interventions. (a) Surface anatomy of the thoracic spine. The ultrasound transducer positions are demonstrated with a yellow rectangle. Scanning from lateral to medial (a; pink arrow), the ribs may be seen transitioning to the thoracic facet joints, then the laminae and facet joints, and finally the spinous processes in midline. The in-plane inferior-to-superior needle trajectory for (a) thoracic intra-articular injection is shown with a blue arrow. The transverse transducer position and in-plane lateral-to-medial needle trajectory is also shown for (b) thoracic medial branch block, at the superolateral aspect of the desired level’s transverse process. (b) Thoracic transverse process, as seen with an ultrasound transducer in saggital orientation. (c) Thoracic facet joints, as seen with an ultrasound transducer in saggital orientation. For intra-articular injection, the needle (blue arrow) is advanced in-plane to the target from an inferior-to-superior trajectory. (d) Thoracic medial branch block. With an ultrasound transducer in transverse orientation, the needle (blue arrow) is advanced in-plane to the target from a lateral-to-medial trajectory. For T5 to T8 medial branch block, the transducer is swept just superior to this view (not shown) and the needle is advance in-plane from a lateral-to-medial trajectory to intertransverse space, at a depth no deeper than the surface of the adjacent transverse processes. ES = erector spinae muscles; IAP = inferior articular process; SAP = superior articular process.

Ultrasound-guided thoracic facet joint intra-articular injection was first described by Stulc and colleagues.^56^ The patient is positioned prone (Figure 4a). To identify the correct level, the 12th rib is first visualized by scanning a sagitally oriented transducer from a caudal-to-cranial direction along a vertical line inferior to the medial border of the scapula. The 12th rib is then followed medially to first show the T12 costotransverse articulation, T12 transverse process (Figure 4b), and then the T12 lamina. The facet joint is seen as a hypoechoic cleft between the respective hyperechoic laminae and articular processes (Figure 4c) and is bounded by the spinous process in the midline. The medial and lateral borders of the facet joint are identified by sweeping the transducer medially and laterally. The mid-point of the joint is typically targeted, using an in-plane caudad-to-cephalad trajectory. Subsequent facet joint levels can be counted by scanning superiorly from T12.

#### Thoracic Medial Branch Block

A technique for ultrasound-guided thoracic medial branch blocks was suggested by Moon et al., though their method has not been validated.^57^ The variable course of the medial branch at different levels of the thoracic vertebrae necessarily entails different bony targets depending on the region of interest (Figure 2). The target level is identified as above, and the transducer is oriented transversely to demonstrate the costotransverse junction (Figure 4a,d). From a lateral-to-medial trajectory, the needle is directed to the periosteum at the superolateral edge of the transverse process, which is the target for T1 through T4, along with T9 and T10.

From T5 to T8, the medial branches do not touch the transverse process but are suspended in the intertransverse space. So, from the superolateral edge of the transverse process, the needle is slightly withdrawn and redirected slightly cephalad. To avoid breaching the pleura, the needle tip is advanced only to a comparable depth as the superficial edge of the transverse process.

The T11 and T12 medial branches have similar anatomical characteristics as the lumbar medial branches, described below.

#### Evidence for Thoracic Facet Interventions

The systematic literature search found one study meeting inclusion criteria.^56^

##### Performance-Related outcomes

Stulc and colleagues performed 20 ultrasound-guided thoracic facet intra-articular injections on a single cadaver, with an accuracy rate of 80%, as per CT imaging.^56^

##### Safety

No studies reported safety outcomes for ultrasound-guided thoracic facet joint interventions.

##### Efficacy

No studies reported efficacy outcomes for ultrasound-guided thoracic facet joint interventions.

### Lumbar Facet Interventions

#### Lumbar Facet Joint Intra-Articular Injection

**Figure 5.**
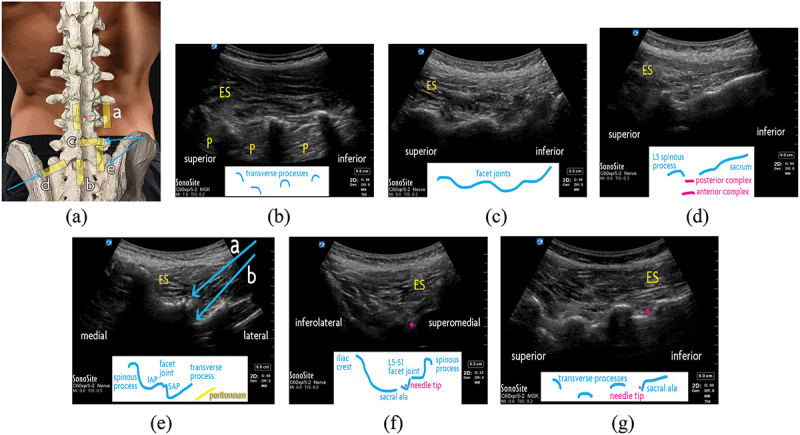
Lumbar facet joint interventions. (a) Surface anatomy of the lumbar spine. The ultrasound transducer positions are demonstrated with a yellow rectangle. Scanning from lateral to medial (a; pink arrow), with the transducer in sagittal orientation, the transverse processes (“trident” sign) may be seen transitioning to the lumbar facet joints (“camel hump” sign), then the laminae, and finally the spinous processes in midline. The sagittal transducer position (b) for examining the lumbosacral junction is also shown. The in-plane lateral-to-medial needle trajectory (c) for lumbar intra-articular injection and medial branch block is shown with a blue arrow. Two methods for targeting the L5 dorsal ramus are demonstrated, via an (d) oblique approach and and via an (e) out-of-plane technique. (b) Lumbar transverse processes, as seen with an ultrasound transducer in saggital orientation. The “trident” sign shows the transverse processes as hyperechoic outlines with shadows where the ultrasound beam cannot pass through. (c) Lumbar facet joints, as seen with an ultrasound transducer in saggital orientation. The superior and inferior articular processes of the facet joints form a continuous hyperechoic “camel hump” line. (d) Lumbosacral junction, as seen with an ultrasound transducer in saggital orientation. In midline, the sacrum is viewed as a continuous hyperechoic line. Superior to it are the L5 spinous process and the L5–S1 interspace. Also visualized are the anterior complex (comprising the posterior aspect of the vertebral body, posterior longitudinal ligament, and anterior dura) and posterior complex (comprising the ligamentum flavum and posterior dura), which surround the intrathecal space. (e) L4 vertebra as seen with an ultrasound transducer in transverse orientation. The in-plane lateral-to-medial needle trajectories are shown for (a) facet intra-articular injection and (b) medial branch block. (f) L5 dorsal ramus block with oblique ultrasound transducer positioning. The needle (pink dot) is advanced out-of-plane with an superolateral-to-inferomedial trajectory. (g) L5 dorsal ramus block, using a “pivot” technique immediately after injecting the L4 medial branch. The ultrasound transducer is placed in a sagittal oblique position to show the L3, L4, L5 transverse processes along with the sacral ala. The needle (pink dot) is advanced out-of-plane to the superior aspect of the sacral ala, by incrementally moving the needle tip from the initial position at the L4 medial branch, at the junction of the L5 transverse process and superior articular process. ES = erector spinae muscles; IAP = inferior articular process; P = psoas muscle; SAP = superior articular process.

Ultrasound-guided lumbar facet joint intra-articular injection was introduced almost two decades ago.^58,59^ The patient is positioned prone (Figure 5a). The transducer is placed on the lumbar region in sagittal orientation. The most lateral structures seen are the transverse processes, in a characteristic “trident” configuration (Figure 5b). The transducer is swept medially, first showing the facet joints (i.e., continuous “camel hump” pattern; Figure 5c) and then spinous processes in the midline. By sliding the transducer caudad, the lumbosacral junction can be dynamically visualized, permitting identification of the desired levels (Figure 5d). The sacrum is a curved, hyperechoic surface appearing continuous except at the sacral foramina. The L5–S1 facet joint is immediately cephalad to the sacral outline, and further levels are counted upwards sequentially.

At the target level, the transducer is rotated transversely to simultaneously show one level’s spinous process, lamina, articular facets, and transverse processes. Rocking the transducer reveals the cleft between the joint’s superior articular process and the inferior articular process (Figure 5e). The needle is advanced in a lateral-to-medial, in-plane trajectory. To verify needle tip placement, the transducer may be rotated sagitally to show the needle tip lying on the middle portion of the facet joint.

#### Lumbar Medial Branch Block

The L1 to L4 medial branches are consistently found at the junction of the transverse process and the superior articular process, which may be found with the transducer oriented transversely at the desired level. The junction appears as a step-like shadow deep and lateral to the facet joint and is targeted via an in-plane lateral-to-medial trajectory (Figure 5e). Rotating the transducer to a sagittal orientation, needle tip placement may be confirmed at the superior aspect of the transverse process prior to injection.

The L5 dorsal ramus contributes to innervation of the L5–S1 facet joint, which is the most commonly involved level in lumbar facet joint-mediated spinal pain. The L5 dorsal ramus, however, is particularly challenging to block due to its depth, obscuration by the iliac crest, and variable sacral anatomy. Greher and colleagues described an early technique to inject this target.^60^ With sagittal transducer orientation, the L5 transverse process and the hyperechoic sacrum are first identified. The transducer then is rotated obliquely, almost 90°, with the medial part more cranial than the lateral part (Figure 5f). In this view, the iliac crest is the most lateral structure. Looking medially, the sacral ala is seen along with the S1 superior articular process; the junction of these two structures is targeted with an in-plane trajectory.

An alternative “pivot” technique for L5 dorsal ramus block begins with the transducer oriented transversely, as seen while targeting the L4 medial branch as described above.^61^ Next, the transducer is rotated sagittally to view the L3–L4 to L5–S1 facet joints and then is swept slightly laterally to show the L3 to L5 transverse processes and sacral ala (Figure 5g). From the junction of the L5 transverse process and superior articular process, the needle is progressively redirected (“walked”) inferiorly and medially down to its target, the junction of the sacral ala and superior articular process. To avoid inadvertently advancing the needle into the L5 foramen, the needle tip must not advance deeper than a line connecting the L5 transverse process and sacral ala.

#### Evidence for Lumbar Facet Interventions

Thirty of the included studies were focused on facet joint interventions, of which 18 were observational studies and 4 were randomized controlled trials (Table 1). Two studies used a combination of different methodological designs.^26,27^

Fourteen studies examined interventions targeting nerves innervating the facet joints (i.e., medial branches, L5 dorsal ramus), and 16 studies assessed facet joint intra-articular injection. There was 1 study of ultrasound-guided medial branch radiofrequency ablation in cadavers^62^ and 1 study of ultrasound-guided medial branch cryoneurolysis in patients.^39^

##### Performance-Related outcomes

Based on fluoroscopic confirmation of needle tip placement, the success rate for ultrasound-guided facet joint intra-articular injection ranged from 86% to 100%.^58,59,63–65^ There was not a consistent advantage in procedural time when ultrasound was compared to fluoroscopy or CT guidance.^59,62,66–68^ In one randomized controlled trial, ultrasound-guided facet joint intra-articular injection was found to be more accurate (86% versus 31%) than a landmark-based technique (i.e., needle entry site at predefined distance from palpated spinous process).^65^

The accuracy for needle tip placement in ultrasound-guided lumbar medial branch intervention was highly variable in both clinical and cadaver studies. In these studies, patients with obesity were often excluded. One observational study, focused on patients with body mass index over 30, reported 62% accuracy on fluoroscopy confirmation and concluded that ultrasound guidance alone in this population is unreliable.^69^ In other studies, the accuracy rate ranged from 72% to 97%.^26–28,39,60,61,63,64,69–76^ The L5 dorsal ramus was identified as a particularly challenging target, owing to its unique and more variable anatomy,^60,69,70^ and techniques for reaching this target continue to be developed.^60,61^

One cadaver study of ultrasound-guided lumbar radiofrequency ablation, using a sophisticated magnetic needle localization system, demonstrated 97% accuracy on fluoroscopy.^62^

##### Safety

There were no major complications observed. Transient minor adverse effects were infrequently observed (e.g., vasovagal reaction, superficial hematoma, pain exacerbation). One observational study reported blood aspiration during 7% of fluoroscopy-guided medial branch injections, without subsequent sequelae.^74^ Blood aspiration was not reported during any ultrasound-guided interventions.

##### Efficacy

Observational studies^77–79^ and randomized trials^26,58,64,66,80,81^ alike attest to comparable reduction in pain scores and disability between ultrasound- and fluoroscopy-guided lumbar facet intra-articular injections with corticosteroid; these benefits persisted through the follow-up period of each study, which tended to be 3 months or shorter. Two randomized trials of lumbar facet joint intra-articular corticosteroid injection compared ultrasound guidance to landmark-based techniques (e.g., needle entry site at a predefined distance from palpated spinous process); one of these studies demonstrated improved pain reduction with ultrasound guidance up to 6-week follow-up,^81^ but the other study found that the superiority of ultrasound guidance did not persist past the immediate postprocedural period.^64^ Though medial branch block is more often used diagnostically rather than therapeutically, some authors reported prolonged analgesia and functional improvement after both ultrasound- and fluoroscopy-guided lumbar medial branch injection with corticosteroid, lasting weeks to months.^71,73^

A case series of satisfactory ultrasound-guided lumbar medial branch cryoneurolysis was reported, with up to 12-month follow-up.^38^

### Future Developments in Ultrasound Guidance

Several studies may be highlighted for their use of novel technology, including fusion imaging (i.e., real-time ultrasound coupled with prior CT or magnetic resonance imaging data)^65,67,82^ and magnetic needle tip tracking.^61^ Sophisticated image guidance systems hold promise to improve the accuracy of ultrasound-guided medial branch targeting, which may permit blockade or radiofrequency ablation. However, few data exist to inform the use of such technology, and further investigation is required before widespread adoption is considered.

## Discussion

This systematic literature search revealed a diverse and rich body of human studies concerning ultrasound-guided facet joint interventions. Cervical facet joint intra-articular injection and medial branch or TON block were particularly amenable to the modality, with favorable accuracy (78%–100%), lower procedural time compared to fluoroscopy or CT guidance, and comparable pain relief. Ultrasound guidance provided excellent accuracy for lumbar facet joint intra-articular injection (86%–100%), whereas medial branch and dorsal ramus block had more variable accuracy (72%–97%); the analgesic effect was comparable to that obtained with fluoroscopy and CT guidance.

### Limitations of Ultrasound-Guided Facet Joint Interventions

Although ultrasound presents an opportunity to refine techniques for managing facet joint–mediated spinal pain, enthusiasm for this imaging modality should be tempered with a realistic understanding of its limitations.

#### Limitations Relative to Fluoroscopy

Compared to fluoroscopy, ultrasound-guided interventions may be more challenging when deeper targets affect needle tip visualization (i.e., lumbar or lower cervical levels). Though some degree of error may be reasonable for facet joint intra-articular injection, the diagnostic specificity of the medial branch block depends on accurate needle tip placement, given that minute local anesthetic volumes are administered. Whereas fluoroscopy permits evaluation of appropriate contrast spread during medial branch block and can exclude intravascular injection, this is not possible using ultrasound alone. Indeed, a recent meta-analysis reported an 11% to 13% absolute risk increase for incorrect needle tip placement using ultrasound compared to fluoroscopic guidance.^83^

Additionally, care must be taken to carefully identify the desired spinal levels for intervention. Fluoroscopy or CT is well suited to showing a wide field of view, but ultrasound displays a relatively limited area, which risks targeting the wrong level.^43,50^ Patients with transitional lumbar anatomy (e.g., sacralized L5) are particularly at risk of misidentification of spinal levels using ultrasound.^13,60^

#### Training Requirements

Interestingly, one study described a statistical model for the acquisition of ultrasound-guided lumbar medial branch block proficiency (“learning curve”) by experienced regional anesthesiologists who did not have prior experience in interventional pain medicine. The model estimated that the procedure would need to be performed more than 47 times to achieve an 85% success rate in the technique for nonobese patients.^76^ This is a challenging learning curve compared to some other ultrasound-guided interventions (i.e., approximately 30 injections to become proficient in sacroiliac joint intra-articular injection).^84^

Ultimately, some clinicians may find the reliability of ultrasound-guided lumbar medial branch block to be unacceptable compared to fluoroscopic guidance using well-established and consistent radiographic landmarks for targeting these nerves.^83^

#### Safety Considerations

Real-time ultrasound has been proposed to theoretically reduce the risk of injury to cervical vascular structures.^85^ Incidentally observed blood vessels may often be found around the lower cervical articular pillars.^43,48^ Nonetheless, no specific safety benefit has been conclusively demonstrated in well-powered reports and, in general, data on clinical outcomes after ultrasound-guided cervical interventions are limited.^86^ Additionally, identified cervical vessels may still be at risk of injury if needle tip visualization is poor during needle manipulation. Errant needle tip movement may also risk nerve root or spinal cord injury; our systematic literature search revealed one case report of spinal cord compression due to hematoma following ultrasound-guided C7 medial branch block.^54^ Ultimately, the effectiveness and safety of ultrasound-guided interventions remain highly operator dependent.

### The Choice of Imaging Modality

Based on our literature search, ultrasound appears to be fairly comparable fluoroscopy or CT guidance for cervical and lumbar facet joint intra-articular injection, at least with respect to accuracy, safety, and clinical efficacy. Though medial branch block appears technically feasible, the higher failure rate for deeper structures (e.g., lumbar or lower cervical regions) may result in more false-negative diagnostic injections and risk unnecessarily precluding otherwise appropriate patients from accessing radiofrequency ablation. Negative ultrasound-guided medial branch blocks may need to be repeated to reduce this risk of false-negative results influencing management; however, this extra step may mitigate ultrasound’s potential benefits of improved access and convenience.

Anatomical factors may also play a role in the choice of imaging modality. For patients with obesity, many structures may be deeper and more difficult to visualize. Additionally, ultrasound-guided facet joint interventions have not been well studied in the presence of spinal instrumentation or unusual anatomy (e.g., severe scoliosis), given that these were exclusion criteria in numerous studies. In these scenarios, it would be worth considering fluoroscopy or CT guidance.

With the exception of one prospective study finding similar efficacy and safety for ultrasound- and CT-guided cervical medial branch radiofrequency ablation,^53^ the study of ultrasound guidance for medial branch radiofrequency ablation has generally been limited to small studies of cadaveric specimens.^52,63^ Additionally, radiofrequency ablation requires the needle tip to be adjacent and parallel to the target medial branch along bony structures, which is generally considered more feasible with fluoroscopy guidance.^12,13,86^

However, there may yet be a role for ultrasound to improve the safety of fluoroscopic- or CT-guided cervical interventions, especially around the cervical facet joints where incidental blood vessels are commonly observed.^33,43,50^ A preprocedural ultrasound scan could reveal vulnerable blood vessels that would not be otherwise seen on fluoroscopy or CT and aid in planning the approach for needle advancement.

Beyond clinical considerations, the choice of imaging modality is influenced by external factors. For instance, in the United States, numerous insurance companies require fluoroscopic or CT guidance for reimbursement of facet joint interventions.^13^ Such restrictions potentially limit the use and ongoing refinement of ultrasound guidance for these interventions.

### Areas for Further Study

This systematic literature search highlights some substantial gaps. For example, there is a conspicuous lack of clinical studies assessing ultrasound-guided thoracic facet joint interventions with respect to procedural outcomes, safety, and efficacy. In general, there is a lack of clinical studies examining thoracic facet joint interventions with any imaging modality,^87^ even though a substantial proportion of thoracic spinal pain is facet joint mediated.

Additionally, ultrasound-guided radiofrequency ablation has been infrequently studied. Ultrasound guidance generally lacks the ability to precisely position a radiofrequency cannula parallel to the path of nerves innervating the facet joints, as currently recommended.^12,13^ Yet, this limitation may potentially be overcome with further procedural refinement and technological advances (e.g., fusion imaging). Interestingly, an ex vivo study reported that the burn characteristics of certain multitined radiofrequency cannulas were potentially favorable for cervical medial branch radiofrequency ablation; however, such a technique has not been clinically studied.^88^

## Conclusion

Techniques for ultrasound-guided facet joint interventions have been developed and improved over the past two decades. Desirable accuracy, safety, and efficacy have been observed in some applications (e.g., lumbar facet joint intra-articular injection). However, some ultrasound-guided techniques remain challenging or impractical (e.g., C7 medial branch or L5 dorsal ramus block), and obesity may present a substantial challenge. For certain interventions (e.g., radiofrequency ablation, thoracic facet joint interventions), there is a paucity of literature to inform practice.

## Supplementary Material

Supplemental Material
