# Supplementary material for: Ultrasound-guided axial facet joint interventions for chronic spinal pain: A narrative review
Source: Can J Pain. 2023 May 17;7(2):2193617. doi: 10.1080/24740527.2023.2193617 (PMC10193888; doi:10.1080/24740527.2023.2193617)
Supplement: Supplemental Material [file UCJP_A_2193617_SM8763.docx]

**Supplement 1.** Literature Search Strategy

**PubMed**

((Zygapophyseal Joint[MeSH Terms]) OR (facet joint[MeSH Terms]) OR (vertebrae[MeSH Terms])) AND ((interventional ultrasound[MeSH Terms]) OR (ultrasonography[MeSH Terms]) OR (injections[MeSH Terms]) OR (pain management[MeSH Terms]) OR (fluoroscopy[MeSH Terms])) AND ((chronic pain[MeSH Terms]) OR (back pain[MeSH Terms]) OR (neck pain[MeSH Terms]))

**Medline**

((exp "Zygapophyseal Joint"/) OR (exp "facet joint"/) OR (exp vertebrae/)) AND ((exp "interventional ultrasound"/) OR (exp ultrasonography/) OR (exp injections/) OR (exp "pain management"/) OR (exp fluoroscopy/)) AND ((exp "chronic pain"/) OR (exp "back pain"/) OR (exp "neck pain"/))

**Cochrane Library**

(([mh "Zygapophyseal Joint"]) OR ([mh "facet joint"]) OR ([mh vertebrae])) AND (([mh "interventional ultrasound"]) OR ([mh ultrasonography]) OR ([mh injections]) OR ([mh "pain management"]) OR ([mh fluoroscopy])) AND (([mh "chronic pain"]) OR ([mh "back pain"]) OR ([mh "neck pain"]))

**CINAHL**

(((MH "Zygapophyseal Joint+")) OR ((MH "facet joint+")) OR ((MH vertebrae+))) AND (((MH "interventional ultrasound+")) OR ((MH ultrasonography+)) OR ((MH injections+)) OR ((MH "pain management+")) OR ((MH fluoroscopy+))) AND (((MH "chronic pain+")) OR ((MH "back pain+")) OR ((MH "neck pain+")))

**EMBASE**

((exp "Zygapophyseal Joint"/) OR (exp "facet joint"/) OR (exp vertebrae/)) AND ((exp "interventional ultrasound"/) OR (exp ultrasonography/) OR (exp injections/) OR (exp "pain management"/) OR (exp fluoroscopy/)) AND ((exp "chronic pain"/) OR (exp "back pain"/) OR (exp "neck pain"/))
